# Landscape of the spliced leader trans-splicing mechanism in *Schistosoma mansoni*

**DOI:** 10.1038/s41598-018-22093-3

**Published:** 2018-03-01

**Authors:** Mariana Boroni, Michael Sammeth, Sandra Grossi Gava, Natasha Andressa Nogueira Jorge, Andréa Mara Macedo, Carlos Renato Machado, Marina Moraes Mourão, Glória Regina Franco

**Affiliations:** 1 Laboratório de Genética Bioquímica, Departamento de Bioquímica e Imunologia, Universidade Federal de Minas Gerais, Belo Horizonte, 31270-901 Brazil; 2 Laboratório de Bioinformática e Biologia Computacional, Coordenação de Pesquisa, Instituto Nacional de Câncer José Alencar Gomes da Silva, Rio de Janeiro, 20231-050 Brazil; 3 Bioinformatics in Transcriptomics and Functional Genomics (BITFUN), Instituto de Biofísica Carlos Chagas Filho, Universidade Federal do Rio de Janeiro, Rio de Janeiro, 21941-901 Brazil; 4 Laboratório Nacional de Computação Científica, Petrópolis, 25651-075 Brazil; 5 Grupo de Helmintologia e Malacologia Médica, Instituto René Rachou, Fundação Oswaldo Cruz, Belo Horizonte, 30190-009 Brazil

## Abstract

Spliced leader dependent trans-splicing (SLTS) has been described as an important RNA regulatory process that occurs in different organisms, including the trematode *Schistosoma mansoni*. We identified more than seven thousand putative SLTS sites in the parasite, comprising genes with a wide spectrum of functional classes, which underlines the SLTS as a ubiquitous mechanism in the parasite. Also, SLTS gene expression levels span several orders of magnitude, showing that SLTS frequency is not determined by the expression level of the target gene, but by the presence of particular gene features facilitating or hindering the trans-splicing mechanism. Our in-depth investigation of SLTS events demonstrates widespread alternative trans-splicing (ATS) acceptor sites occurring in different regions along the entire gene body, highlighting another important role of SLTS generating alternative RNA isoforms in the parasite, besides the polycistron resolution. Particularly for introns where SLTS directly competes for the same acceptor substrate with cis-splicing, we identified for the first time additional and important features that might determine the type of splicing. Our study substantially extends the current knowledge of RNA processing by SLTS in *S. mansoni*, and provide basis for future studies on the trans-splicing mechanism in other eukaryotes.

## Introduction

In contrast to conventional splicing in cis, trans-splicing connects exons of two different primary RNA molecules transcribed from *a priori* unrelated genomic *loci*. One prominent variant of trans-splicing, the spliced leader (SL) dependent trans-splicing (SLTS), has been reported in several organisms^1^, including the flatworm *S. mansoni*, the etiologic agent of schistosomiasis. Both cis- and trans-splicing processes are catalyzed by the spliceosome, a large dynamic complex composed by U-rich small nuclear RNAs (U snRNAs) associated with Sm proteins originating RNA-protein complexes called small nuclear ribonucleoproteins (snRNPs). In SLTS, however, the SL RNA splicing substrate itself is assembled into a core snRNP by directly binding to the Sm proteins, thereby replacing the snRNA U1 function in cis-splicing^2,3^. Additionally, unlike cis-spliceossomal counterparts, the SL RNP is consumed during the trans-splicing reaction: the SL exon harboring the 5′ splice donor is joined to the 3′ splice acceptor sequence in the target RNA molecule. SLTS in *S. mansoni* depends on a specific 36nt SL exon derived from a non-polyadenylated 90nt long precursor^4^ and the SLTS mechanism is not directly associated with a specific gene category, subcellular localization or life-cycle stage^5–7^.

The best-characterized function of SLTS is the resolution of polycistronic transcripts into monocistronic RNA molecules^8–11^. However, in several organisms, most of the known trans-spliced mRNAs are produced from monocistronic RNAs, underscoring that SLTS exert other important genomic functions such as: to increase transcript stability and translational efficiency by adding a specialized cap to mRNA (reviewed in^12,13^); to permit plasticity of 5′ gene structures^14^. Furthermore, SLTS can also regulate gene expression by substituting start codons during outron removal^15^ and specifically in flatworms, SL trans-splicing can provide the start codon for translation of an mRNA^16^.

The current knowledge on SLTS mechanisms suggests that the trans-splice acceptor is preferably located within the 5′-region of the first exon of a target gene. This is based on the premise that the presence of additional cis-splicing signals in the outron, such as upstream donor sequences, would disturb the establishment of the SLTS process (reviewed in^13^). However, recent reports have showed that SLTS occurs at distal sites of the 5′-end of transcripts^5,15^, including 3′ splice sites of long introns^17^, suggesting that the trans-splicing machinery can also interpret other transcript regions as outrons. Additionally, it has been reported the existence of intronic promoters in large introns that harbor alternative SL acceptor sites and that these independent promoters, together with SLTS, can provide tissue specificity for gene expression in *Caenorhabditis elegans*^18^. Moreover, in *Ciona intestinalis*, a substantial proportion of SLTS splice acceptors exhibit adjacent cryptic target sites, suggesting the existence of alternative trans-splicing (ATS), in analogy to commonly studied models of alternative cis-splicing (ACS)^19^.

Organisms such as *S. mansoni*, where cis and trans-splicing co-exist, offer a challenging opportunity to understand how these mechanisms are coordinated. Also, although important aspects of the SLTS mechanism in *S. mansoni* have been elucidated^5,7,20^, most of the previous studies have evaluated a small portion of the total SLTS transcripts repository, yielding limited information about the SLTS mechanism and underestimating the true impact of this phenomenon on schistosome biology. Therefore, using the SL Trapping methodology^15^, we performed a deep and comprehensive analysis of the *S. mansoni* trans-spliced transcriptome. In this work, we aimed at decrypting the SLTS mechanism and assessing the importance of trans-splicing as a regulatory mechanism in this parasite. Here, we characterize the SLTS as a ubiquitous regulatory mechanism in *S. mansoni* and demonstrate for the first time widespread ATS acceptor sites occurring along the entire transcript body of target genes. Most importantly, we identify specific transcript features that may determine the decision as to whether to use a 3′ splice site for cis or trans-splicing.

## Results

### Comprehensive landscape of the SLTS mechanism generated by directed capture of SL-containing transcripts

**Figure 1:**
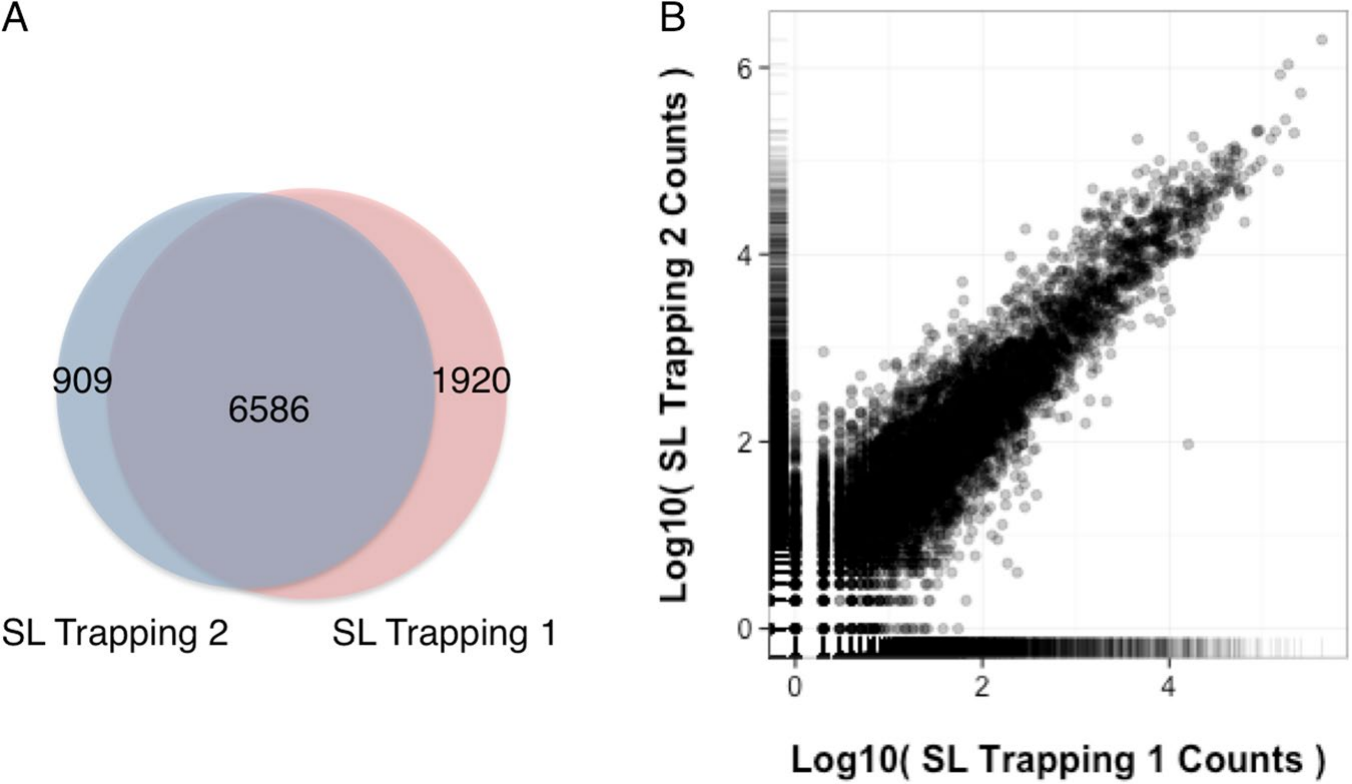
Comparison between the two SL Trapping experiments. (**A**) Shared genes between the two independent biological replicates, SL Trapping 1 (pink) and SL Trapping 2 (blue). (**B**) Correlation of gene counts between the two SL Trapping datasets (Pearson correlation coefficient = 0.86).

To explore the role of SLTS in *S. mansoni*, we performed two experiments of SL Trapping by targeting transcripts containing the SL sequence from cercariae, a life-cycle stage of the parasite (herein referred to as “Trapping 1” and “Trapping 2” datasets with 11,520,178 and 30,332,894 reads, respectively). From these data, 34,929,746 reads (83%) were uniquely mapped to the *S. mansoni* reference genome (5^th^ version). Additionally, a total of 9,415 genes were identified in both Trapping datasets, demonstrating a clear reproducibility between the two biological replicates: 70% (6,586) overlap between Trapping 1 and Trapping 2 datasets (Fig. 1A). Moreover, we observed a strong correlation between the frequencies of the SLTS events in transcripts present in both datasets (0.86 Pearson correlation coefficient - PCC, Fig. 1B).

In order to assess the accuracy of the SLTS targets identified in the Trapping datasets, we compare them with the targets found using an *in silico* approach to recover SL containing reads from publicly available RNA-Seq datasets (whole-transcriptome). We retrieved 91,188 reads harboring the 36-nucleotide SL sequence from approximately 250 million reads comprising experiments with different *S. mansoni* life-cycle stages, herein referred to as the RNA-Seq Filtered dataset. Transcripts undergoing SLTS were rare in the RNA-Seq dataset (SL sequences were detected in <0.04% of the total RNA-Seq reads), and accordingly, we only retrieved 2,459 SL-containing transcripts from the whole-transcriptome RNA-Seq datasets. Despite their distinct origins, 95% of the trans-spliced transcripts observed in the RNA-Seq Filtered dataset were also detected in both Trapping datasets (Supplementary Fig. S1). The majority of the remaining 5% (123 transcripts) are probably expressed specifically on the two life-cycle stages (schistosomula and adult worms) represented in the RNA-Seq Filtered dataset, but not in the Trapping datasets. In fact, only 22 transcripts exclusively observed in the RNA-Seq Filtered dataset are expressed in cercariae (Supplementary Fig. S1).

For further analyses, we curated the SL trapping dataset and considered only gene transcripts identified with at least 10 read counts in both Trapping replicates. From 9,415 transcripts, 5,443 were selected (Supplementary Fig. S1), which represent approximately 58% of the total gene transcripts identified by this approach. Despite the heterogeneous origins of the experimental material (cercariae versus cercariae, schistosomula and adult mixed genders) and different methodological strategies, the final Trapping datasets cover approximately 85% of the genes identified on the RNA-Seq Filtered dataset (Supplementary Fig. S1). Interestingly, 2,081 gene transcripts shared by both the Trapping and RNA-Seq Filtered datasets are frequently trans-spliced genes (507 average read counts for the genes shared by the Trapping and Filtered datasets versus 44 average read counts for the genes exclusively detected in the Trapping datasets), which reinforces the sensitivity of the SL trapping strategy as a method for capturing rare trans-spliced transcripts (Supplementary Fig. S1). Quantitatively, the Trapping and Filtered datasets exhibit a moderate correlation (PCC = 0.5, Supplementary Fig. S1), and in general, the gene transcripts identified in both datasets share the main SL insertion sites.

By assessing the frequency of SLTS events, we identified 5,821 SL-containing transcripts in cercariae, representing 46% of the 12,659 expressed transcripts in this stage (according to RNA-Seq data evaluation, retrieved from public databases) (Supplementary Fig. S1). The proportion increases to 63% when only protein-coding genes are considered (5,062 trans-spliced gene transcripts from a total of 7,997 protein-coding genes expressed in cercariae). Among the trans-spliced transcripts identified, 759 are putatively novel genes (of which 20% are also found in the RNA-Seq Filtered dataset) because they do not match any currently annotated gene model.

**Figure 2:**
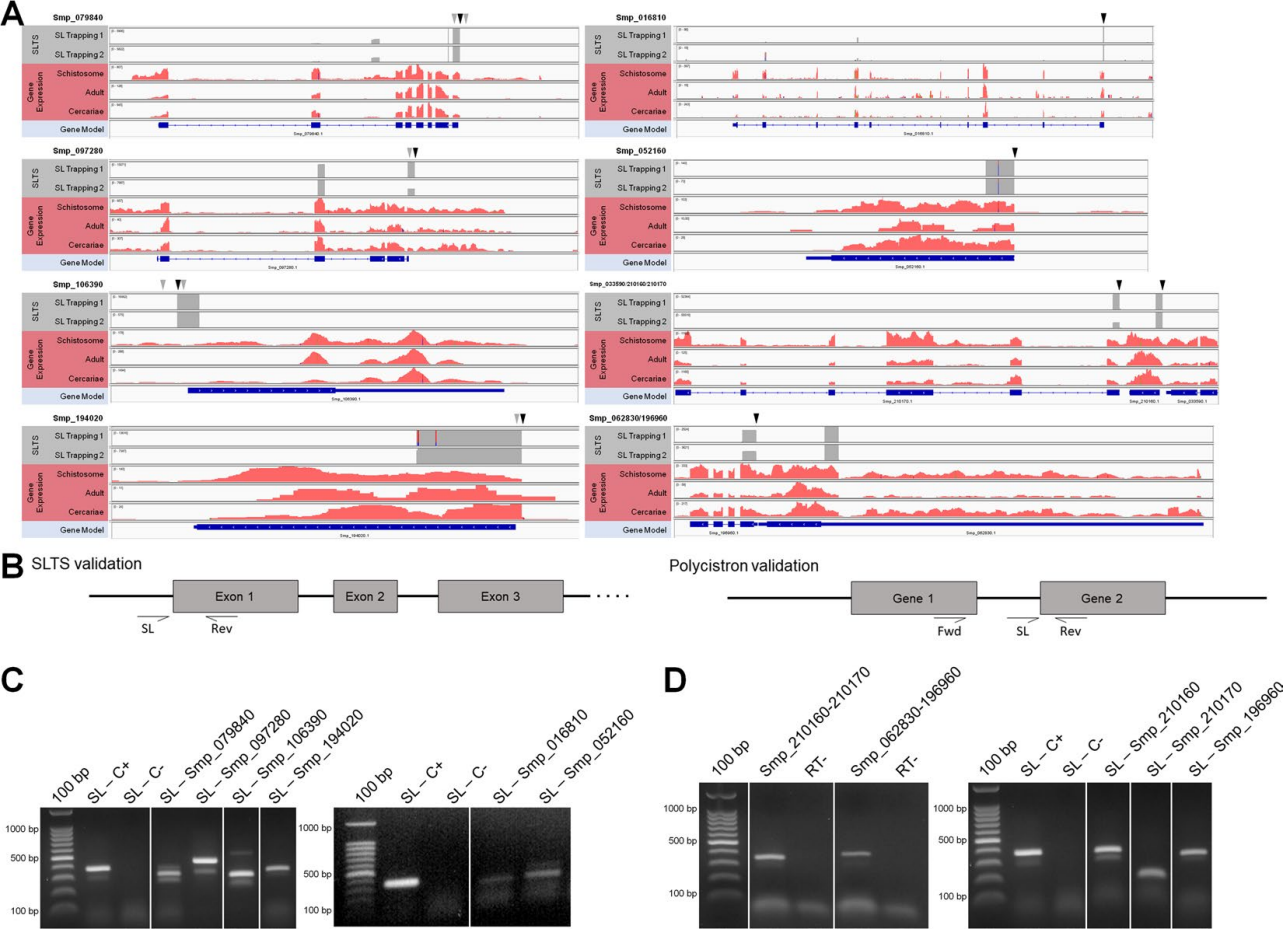
SL insertion sites and validation of novel trans-spliced transcripts. (**A**) Genome browser view of the exon-intron structure of genes undergoing SLTS, with the superimposed coverage for the SL Trapping 1 and 2 datasets (gray - top tracks) and the RNA-Seq dataset (pink - middle tracks). Black arrows show SL insertion sites and gray arrows show secondary SL insertions sites. The structure of genes with high (Smp_079840, Smp_097280, Smp_106390 and Smp_194020) and low read counts (Smp_016810 and Smp_052160), as well as the gene structure of a tricistronic transcript constituted by the genes Smp_033590, Smp_210160 and Smp_210170, and a dicistronic transcript, constituted by the genes Smp_062830 and Smp_196960, are shown. (**B**) Schematic view of genes validated by RT-PCR with the primers annealing sites. The expected fragment sizes were described in Table S1. (**C**) RT-PCR validation of trans-spliced genes with SL as forward primer and a gene-specific reverse primer. The Smp_024110 and Smp_045200 genes were included as positive and negative control, respectively, previously reported in^20^. (**D**) RT-PCR validation of two polycistrons by two PCR reaction: with SL as the forward primer and a gene-specific reverse primer and with gene specific primers from upstream and downstream genes. Controls without reverse transcriptase were used for each of the reactions to show the absence of gDNA contamination. Full-length gels are presented in Supplementary Figure S5 A, B and C.

To validate the power of the SL Trapping approach in revealing new trans-spliced transcripts in *S. mansoni*, we evaluated by RT-PCR the expression of cercariae transcripts represented in the Trapping dataset with high read counts (Smp_079840, Smp_097280, Smp_106390 and Smp_194020) and low read counts (Smp_016810 and Smp_052160) (Fig. 2). In all cases amplifications were made by using the SL sequence as forward primer and a gene-specific reverse primer designed for the first exon of the gene (Table S1A). RT-PCR results from Fig. 2 reveal that more than one amplification product was generated for some transcripts, evidencing another important feature of this mechanism: the presence of alternative trans-splicing sites (ATS) nearby the main STLS entrance (as an example, the transcript Smp_079840 presents 3 different acceptor sites in the outron region). Another important observation is that the secondary SL entry sites can be either upstream or downstream to the main site and there is no fixed distance between the secondary and the main sites. For most cases presented here we could identify reads mapping to the ATS, but with lower counts than the main site. However, it was not possible to map any read from the SL Trapping libraries upstream to the main acceptor site of the gene Smp_106390 to explain the larger band observed in the gel. The reason for it was probably because this gene is annotated in the beginning of a contig, which could impair the mapping of reads to this region.

### Features of the SLTS mechanism in *S. mansoni*

In dinoflagellates, certain genes present multiple SL sequences in tandem, which are probably derived from the reverse transcription of previously trans-spliced transcripts and the subsequent reinsertion of the corresponding cDNA in the genome^21^. After scanning for SL sequences in the *S. mansoni* genome, we detected 32 extra *loci* in addition to the five SL RNA genes annotated in the 5^th^ version of the *S. mansoni* genome and seven non-annotated putative full-length SL RNA genes (93.3 to 97.8% identity with the 90 nt SL RNA and 90.3 to 100% identity with the 36 nt SL exon sequence). These new *loci* include either full or partial exonic SL sequences of which 11 are merged to previously annotated genes. After re-annotation of the *S. mansoni* transcriptome, we found six exonic SL sequences merged to transposable elements (Supplementary Table S2). Seventeen genes that contain the SL exon are not currently annotated, which indicates the existence of additional unknown genes in the *S. mansoni* genome. The aforementioned genes containing genome-encoded exonic SL sequences were discarded throughout subsequent analyses of the SLTS mechanism.

Next, we investigated whether certain *S. mansoni* trans-spliced transcripts are originated from the processing of polycistronic units. We found 139 candidate operons (gene clusters with intergenic distances of up to 200 bp, as specified in^20^), of which only 66 showed evidence of SLTS in the SL Trapping dataset. We thus identified 65 *bona fide* dicistronic units and one tricistronic unit, including 34 of the 46 previously identified polycistrons^20^ (Supplementary Table S3), containing the SL sequence as evidence that they were transcribed and trans-spliced. We validated the expression of two new polycistronic transcripts through RT-PCR, by amplifying the intergenic region between two cistrons^20^ (Fig. 2 and Supplementary Table S1). For the dicistron Smp_062830 - Smp_196960 we amplified the intergenic region (no amplification was seen when no reverse transcriptase was added to the reaction) and also the trans-spliced second cistron. For the tricistron Smp_033590 - Smp_210160 - Smp_210170, in addition to the independent amplification of the two last cistrons using the SL as forward primer and a gene-specific reverse primer, we also amplified the intergenic region between Smp_210160 and Smp_210170, demonstrating the existence of a polycistronic transcript containing, at least, the second and third genes of the tricistron. Attempts to amplify the region between Smp_033590 and Smp_210160 were not successful perhaps because the expression of the first gene of this tricistron is lower (see read counts in the SL Trapping dataset at Table S3) than the expression of the last two cistrons. Although we could not validate by RT-PCR the expression of the first intergenic region of the polycistronic unit, reads from total RNA-seq can be found mapped to this intergenic region, especially in the schistosome stage. We believe that probably the trans-splicing reaction to remove the first intergenic region is faster than the removal of the second region, which may hamper the amplification of this region. This can also be explained by the higher score obtained for the upstream acceptor site used to resolve the first and second cistrons when compared with the downstream acceptor site to resolve the second and third cistrons (geneID score of 4.81 versus 3.71), since the conservation of splicing motifs is related to the affinity for splicing factors.

Most polycistronic units identified in this study encode conserved genes and 23% are monoexonic transcripts; 131 are protein-coding genes and two are rRNAs. Polycistrons are predominantly located at chromosome 1 (25.75% of all polycistrons), chrW (22.72%) and chr4 (15.15%), in contrast to the monocistronic genes undergoing trans-splicing, which are homogeneously distributed along the whole genome. In the polycistronic gene pairs, we could not find a correspondence between expression levels of the partner genes, suggesting that additional post-transcriptional mechanisms may regulate their levels in the cell. Although our observations are probably a low estimate of the actual number of polycistronic clusters, because polycistron gene components with incomplete 5′ or 3′ transcript annotations, that are separated by unusually large spacers, or not expressed in our dataset, most likely fall under the detection threshold of our analysis, the 133 polycistronic gene components accounted only for 2% of the 5,821 transcripts undergoing trans-splicing, implying another important regulatory role for SLTS in the parasite apart from polycistron resolution. Our observations contrast with previous studies in *C. elegans* and *C. intestinalis* wherein 15%^17^ and 20%^19^, respectively, of all trans-spliced transcripts are attributed to operons resolution.

To further analyze the characteristics that discriminate genes subject to SLTS from all protein-coding genes expressed in the cercariae stage, we analyzed the Gene Ontology (GO) biological processes of 5,061 protein-coding genes expressed in the Trapping datasets and compared to the 7,997 protein-coding genes expressed in cercariae (data from^20^). By employing a clustering algorithm to select for enriched gene functional categories, we found that, in general, the different categories for the trans-spliced genes do not differ significantly from all protein-coding genes expressed in cercariae, except in the “Unknown” and the “Transcription factor” functional categories (p-value < 0.024 and p-value < 0.0003, respectively, Chi-square test; Supplementary Fig. S2). Additionally, trans-spliced gene transcripts did not exhibit significant enrichment in any specific Kyoto Encyclopedia of Genes and Genomes (KEGG) pathway^22^, suggesting that SLTS-derived gene products act evenly on essential *S. mansoni* metabolic pathways (Supplementary Fig. S3).

### Attributes of trans-spliced gene transcripts

**Figure 3:**
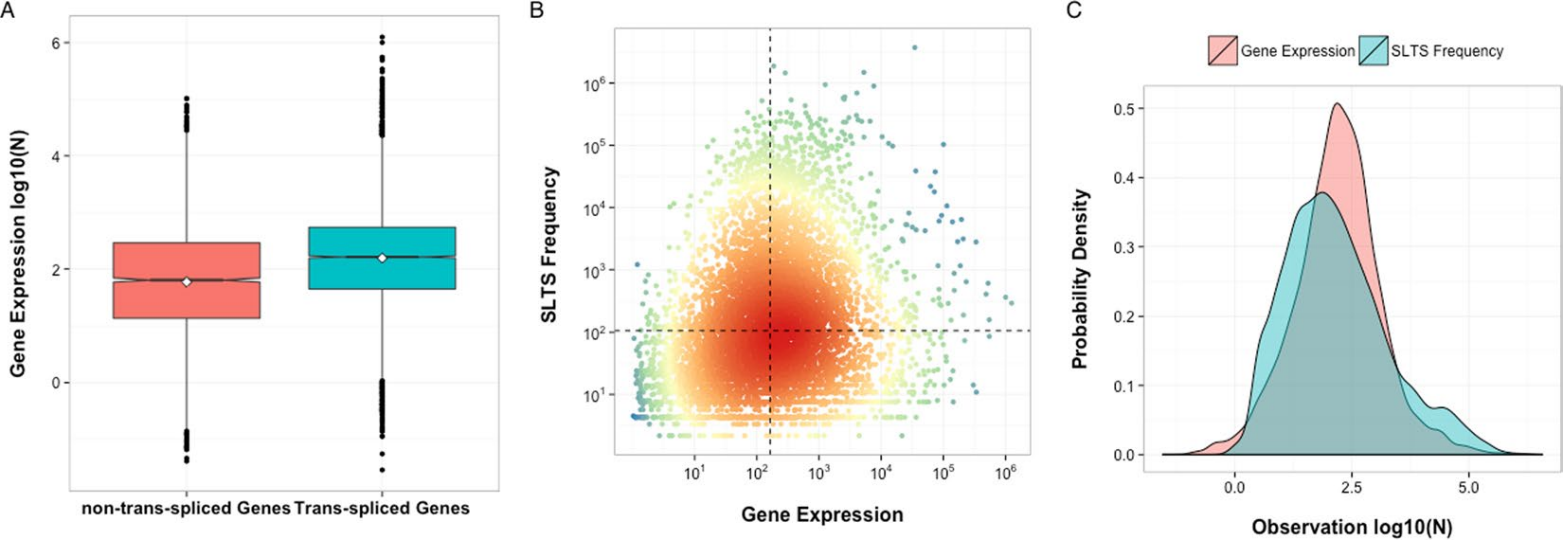
Relationship between gene expression and trans-splicing frequency. (**A**) Normalized log-expression levels of non-trans-spliced genes (pink box) compared with the log-expression levels of trans-spliced genes (blue box), p-value < 1.3e-08, two-sample t-test. (**B**) Normalized expression for the trans-spliced genes is compared with the normalized SLTS frequency. Dashed black lines denote the average levels of both indicators (163 for gene expression and 106 for SLTS frequency), segregating the genes with relatively low SLTS frequency (lower-right quadrant) from genes with relatively high SLTS frequency (upper-left quadrant) and comparing their expression levels (rank = 0.208, Spearman correlation coefficient). (**C**) The probability distribution of the gene expression levels (pink curve) and SLTS frequencies (blue curve) (p-value < 0.11, Kolmogorov-Smirnov).

By accessing a *S. mansoni* cercariae whole transcriptome data^20^ we obtained the total expression levels of all trans-spliced gene transcripts identified in this study and further evaluated the correlation between gene expression and SLTS occurrence. We observed a tendency to the high expression of genes undergoing SLTS, since the median normalized expression value of the trans-spliced genes, according to the RNA-Seq data, is approximately 5-fold greater than the expression of non-trans-spliced genes (161.2 versus 32.3, p-value < 1.3e-08 in a two-sample t-test, Fig. 3A). This observation raises a question whether the gene expression levels determine SLTS frequency (highly expressed genes are more efficiently trans-spliced) or genes that are less expressed will be less likely to be detected by the SL Trapping strategy. However, we can see a weak correlation between gene expression and SLTS frequency (rank = 0.208, Spearman correlation coefficient). We observed in our dataset highly expressed genes that are rarely trans-spliced, and vice-versa (Fig. 3B, lower-right and upper-left quadrants, respectively), indicating that these findings are not a methodology bias, since the trapping methodology is capable of effectively detect trans-splicing even in low expressed genes, and also suggesting that the SLTS process is not exclusively determined by the gene expression levels. Our analysis also revealed log-normally distributed gene expression levels for the overall transcriptome (pink histogram in Fig. 3C), nevertheless the frequencies of transcripts undergoing trans-splicing deviate from a log-normal distribution (p-value < 0.11 in a two-sample Kolmogorov-Smirnov Test, blue histogram in Fig. 3C). These results suggest that the frequency of SLTS could be influenced by the presence of particular gene features facilitating or hindering the trans-splicing mechanism.

**Figure 4:**
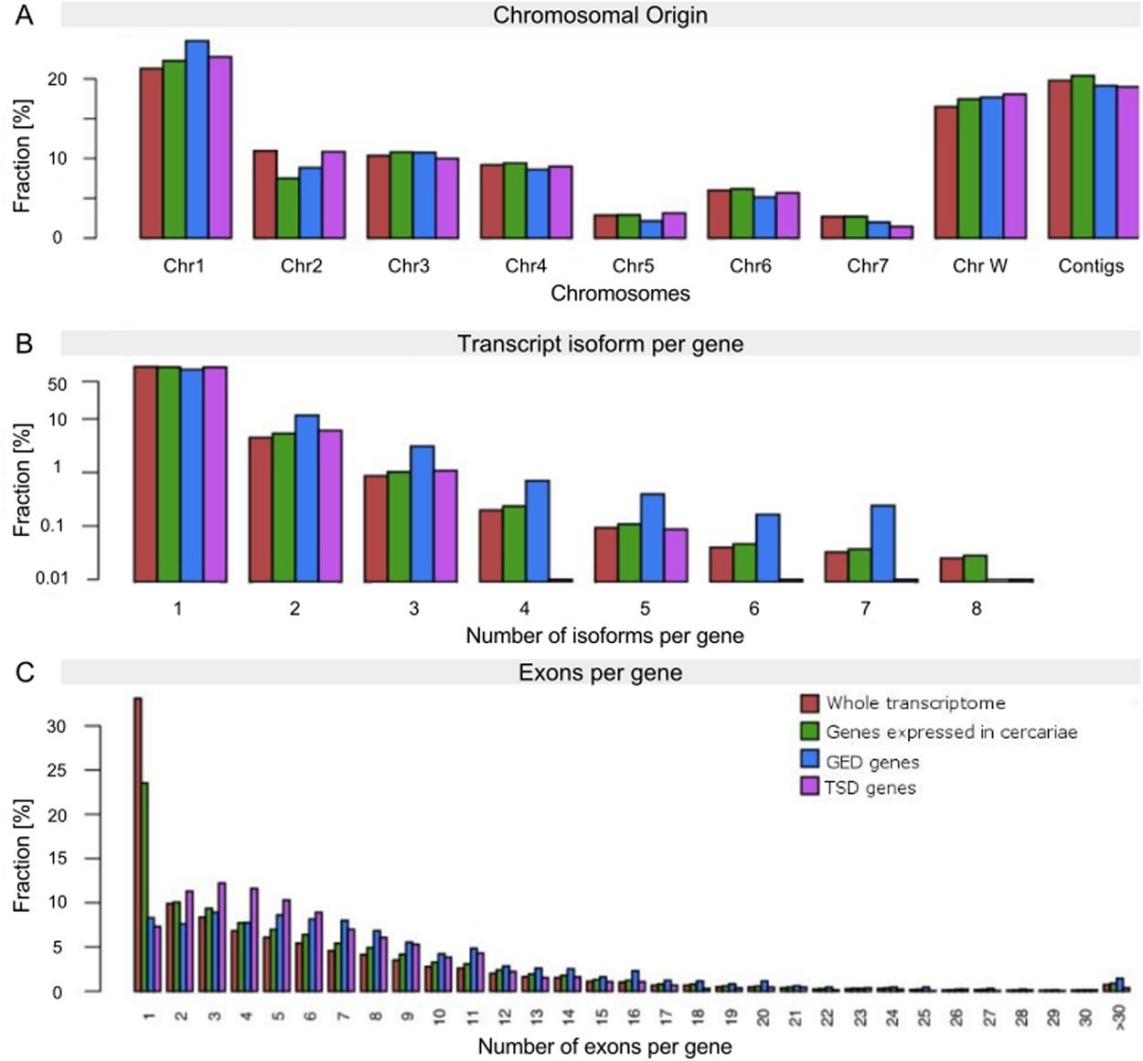
Chromosomal origin, alternative splicing isoforms and number of exons in the trans-spliced genes. (**A**) The relative distribution of trans-spliced genes with high expression levels (GED, blue bars) and trans-spliced genes with low expression levels (TSD, purple bars) in each chromosome is compared with the chromosome distribution of genes expressed on the *S. mansoni* whole transcriptome (red bars) and genes expressed only in the cercariae transcriptome (green bars). (**B**) The relative distribution of alternative splicing transcript isoforms per gene in the *S. mansoni* whole transcriptome, in the cercariae transcriptome, in the subset of GED genes and in the subset of TSD genes. (**C**) The relative distribution of transcripts from genes with single or multiple exons in the *S. mansoni* whole transcriptome, in the cercariae transcriptome, in the subset of GED genes and in the subset of TSD genes.

To further investigate the connection between gene expression and SLTS frequency, we investigated the features that discriminate genes with high expression and low frequency of SLTS events (gene-expression-driven – GED) from genes with low expression and high frequency of SLTS events (trans-splicing-driven – TSD). We selected 2,400 outliers (1,200 outliers from the upper-left quadrant and 1,200 outliers from the lower-right quadrant of Fig. 3B). We observed no significant preferences for a certain chromosomal location in either GED or TSD genes compared with the overall gene expression by chromosomes (Fig. 4A).

When analysing the *S. mansoni* whole transcriptome, we observed that most genes produce a single or few isoforms by alternative cis-splicing (ACS), and only few genes generate multiple transcript isoforms according to the *S. mansoni* GeneDB gene annotation (5^th^ version). A similar finding was seen for GED genes, although for this category a higher number of genes tend to be expressed as different isoforms when compared to the overall gene category. In contrast, we observed significantly less ACS in TSD genes (p-value = 0.08, two-sample Kolmogorov-Smirnov Test), most of which exhibiting no more than three alternative isoforms per gene (Fig. 4B). Similarly, most *S. mansoni* transcripts were derived from genes with one or few exons (Fig. 4C). GED and TSD include fewer single-exon genes and more genes with a moderate to high number of exons than the entire transcriptome (p-value = 0.0005 for both, GED and TSD genes, Kolmogorov-Smirnov Test). Interestingly, while the higher number of exons in GED genes correlates to more ACS isoforms, TSD genes include fewer alternative transcripts compared to the number of exons, which suggests a higher SLTS frequency in the absence of strong ACS-driving signals.

### Comparisons between SLTS and cis-splicing

**Figure 5:**
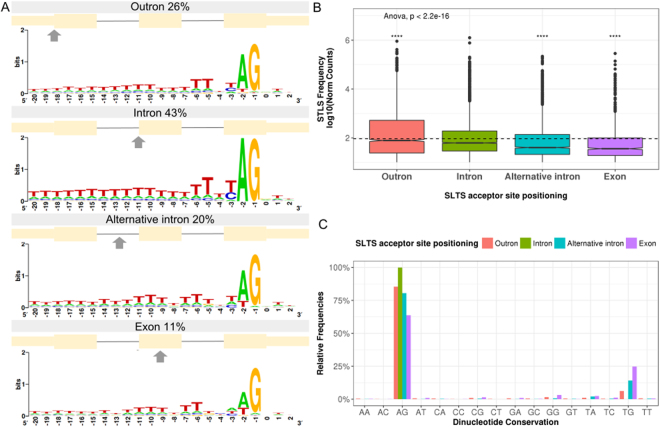
Comparison between different positioning of acceptor sites in outrons and in introns subject to SLTS. (**A**) Relative number of cases and sequence logos of predicted acceptor sites in different positions according to the gene annotation. The positions of acceptor sites resulted from the mapping of SL Trapping reads. SLTS acceptor sites occurring in the first exon of transcripts, in the intron-exon junction, in the intron but upstream to the main acceptor site or located in the exon were named as outron, intron, alternative intron and exon, respectively. (**B**) Quantitative SLTS frequency in each of the different acceptor sites. ANOVA followed by pairwise t-test: ****p < = 0.0001. (**C**) Distribution of the dinucleotides found in each SLTS acceptor site positioning (pink bars - outron, green bars - intron, blue bars - alternative intron and purple bars - exon).

In contrast to the classical notion that the presence of an acceptor site lacking the 5′ splice donor counterpart is the unique SLTS substrate^23^,  a more conservative analysis, where only SLTS acceptor sites identified with 10 reads or more were included, allowed the observation of 6,959 SLTS sites located downstream of the annotated transcription start sites. Although the 5′-end transcript region (outron) in fact constitute the main SLTS substrate, we observed that this corresponds to only 26% of all SLTS events (Fig. 5A), highlighting the plasticity and heterogeneity of alternative SL insertion sites among transcripts. A quantitative analysis in the SL Trapping dataset confirmed that SLTS events occur significantly more frequently in the first exon, which is demonstrated by the note of significantly greater mean read counts associated to the acceptor sites located in the outron region (p-value < 2.2e-16 in a pairwise t-test, Fig. 5B). However, 43% of the SLTS acceptor sites were located at cis-splicing acceptor sites in introns/exon boundaries (Fig. 5A), and for some transcripts, the frequency of SLTS in this region was higher than in the outron. Interestingly, we also observed that sites located in introns/exons boundaries exhibit higher sequence conservation compared with SLTS acceptor sites in the outron (Fig. 5A). Remarkably, the frequency of canonical AG dinucleotides in the outron is lower compared to cis-splicing acceptor sites used for SLTS. Another dinucleotide (TG) is also observed at this position for 6% of the cases (Fig. 5C). Previous reports on *Trypanosoma brucei* showed that 20% of the weak trans-splicing acceptor sites contained a dinucleotide other than AG. The dinucleotide GG occurred in 7% of these sites, while TG, AA, GA and AC were observed in 2% of the weak splice sites^15^. For *S. mansoni*, weaker acceptor sites, as the ones found inside of exons, present only 64% of AG, followed by 25% of TG and 3% of GG (Fig. 5C).

**Figure 6:**
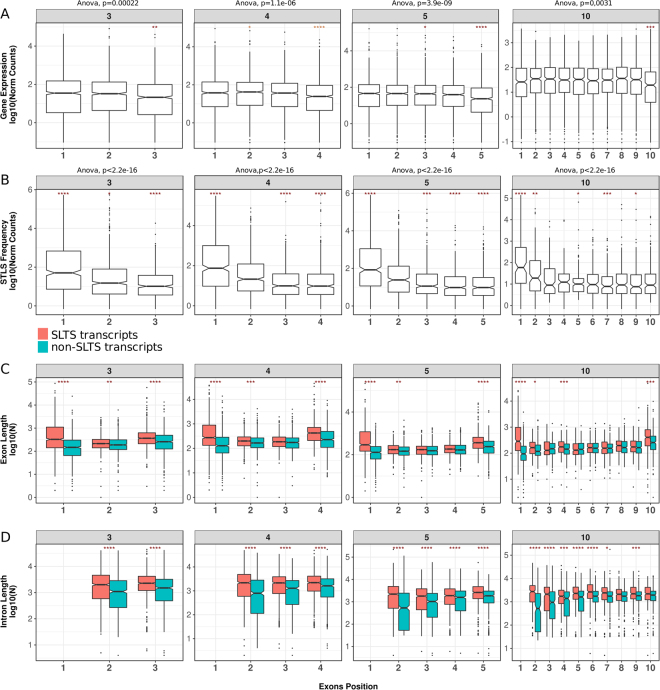
Trans-splicing occurrence by exons position in gene bodies and exon and intron lengths. (**A**) Expression levels of exons according to their position in transcripts (estimated from the RNA-Seq public dataset). (**B**) Frequency of trans-splicing according to exon position (estimated from SL Trapping). For A and B groups of genes were divided according to their number of annotated exons. Only genes with three, four, five and ten exons are shown. Analysis of variance (ANOVA) were performed followed by pairwise t-test (*p < = 0.05, **p < = 0.01,***p < = 0.001, ****p < = 0.0001). For a better visualization, pairwise differences were also tested by Tukey’s Honest significant difference test and the results are plotted on Figure S4. (**C**) Distribution of exon lengths in trans-spliced (pink boxes) and non-trans-spliced (blue boxes) genes that present three, four, five and ten exons. (**D**) Distribution of introns lengths immediately upstream of the splicing event in trans-spliced (pink boxes) and non-trans-spliced (blue boxes) genes presenting three, four, five and ten exons. For data in C and D, t-test were performed: *p < = 0.05, **p < = 0.01,***p < = 0.001, ****p < = 0.0001.

In a distinct analysis, grouping SLTS events by exon position within transcripts revealed a significant preference of SLTS for exons located on the 5′ end of the transcript, as already expected (Fig. 6B and Supplementary Fig. S4, p < 0.0001, pairwise t-test). In parallel, we did not observe higher levels of expression of the initial or final exons of transcripts (Fig. 6A and Supplementary Fig. S4), but, conversely, a significantly depletion in the coverage of terminal exons was seen, which can be explained by the RNA-Seq 3′ bias^24^, reinforcing that the SLTS preference for the first exon is not particularly affected by artefacts produced by the unequal exon expression in RNA-Seq data. We further found that the initial and terminal exons are significantly longer in the transcripts undergoing SLTS (Fig. 6C, p < 0.0001, t-test) and that the introns from trans-spliced transcripts tend to be slightly longer than their non-trans-spliced counterparts (Fig. 6D, p < 0.001, t-test).

For *C. elegans*, it was suggested the two-promoter hypothesis^18,25^, where both internal SL1 entries and alternative transcription initiation from independent protomers embedded in long introns lead to the expression of alternative transcripts that can be related to tissue specificity. In order to check whether a similar mechanism would be occurring in *S. mansoni*, we investigated if the SLTS events occurring in long introns are related to the presence of alternative internal promoters. We performed an *in silico* genome-wide approach to predict promoters and evaluated the enrichment of SLTS acceptor sites downstream to alternative promoters. Putative canonical promoter regions were identified by the consensus predictions of two different tools. From a total of 29,866 promoters predicted by both tools in the *S. mansoni* genome, 1,897 were located in intronic regions and could act as alternative promoters. Out of the 6,071 SLTS acceptor sites occurring in introns, only 561 sites were downstream to the alternative predicted promoters satisfying our criteria. These results indicate that some SLTS events occurring in intronic regions could, in fact, occur in outron regions of transcripts expressed from the alternative promoter present inside the intron, resulting in a primary transcript lacking the cis-splicing donor site. Although our analysis does not account for non-canonical core promoters, the vast majority of alternative SLTS events in intronic regions seems to occur in the presence of the cis-splicing donor site, suggesting a competition between cis and trans-splicing.

**Figure 7:**
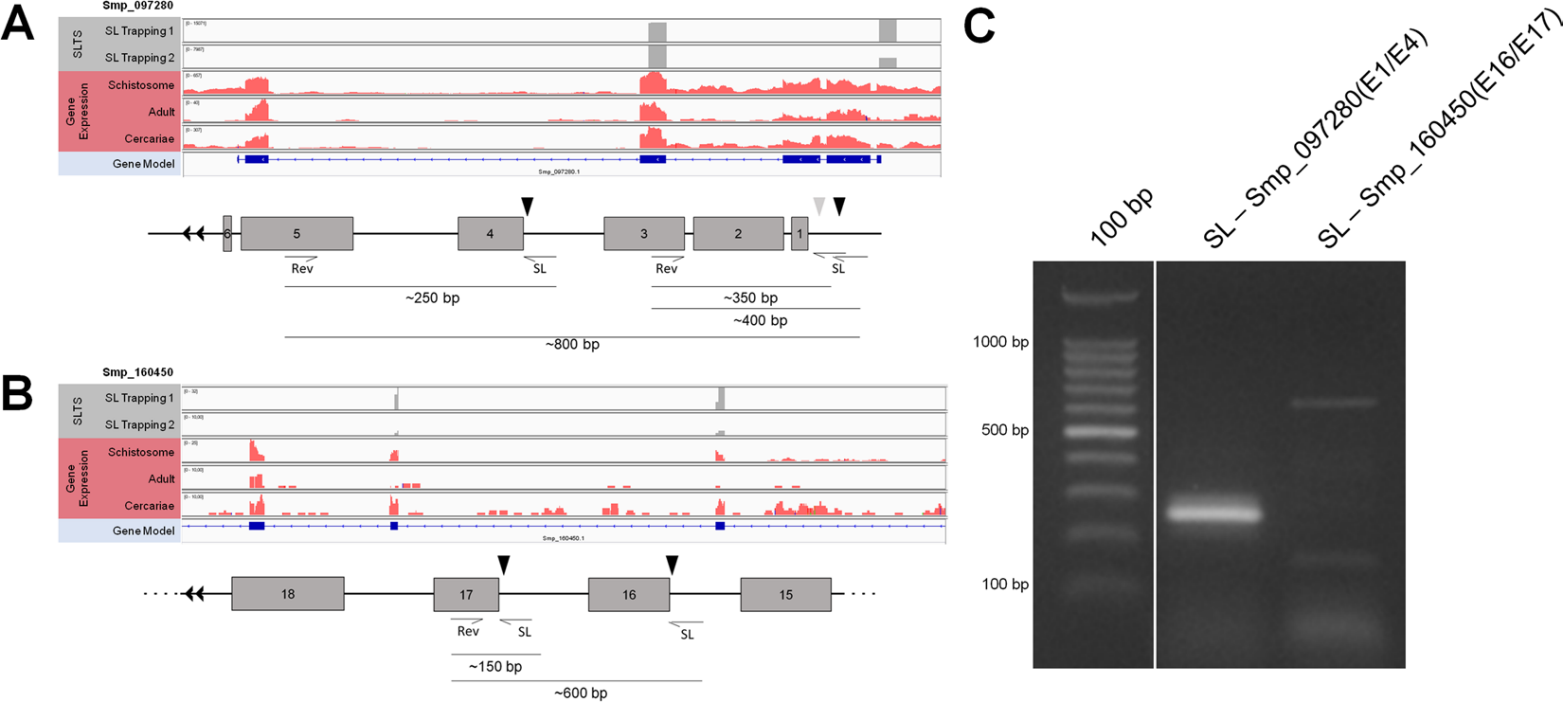
RT-PCR validation of internal trans-spliced genes. Genome browser and schematic view of the exon-intron structure of the genes Smp_062830 (**A**) and Smp_196960 (**B**) undergoing SLTS, with the superimposed coverage for the SL Trapping 1 and 2 datasets (top tracks) and the RNA-Seq dataset (middle tracks). Black arrows show the SL insertion sites and gray arrows show secondary SL insertion sites. The expected fragment sizes are described in the schematic representations. (**C**) RT-PCR validation of internal trans-splicing in the case studies using SL as forward primer and an exon-specific reverse primer. Full-length gels are presented in Supplementary Figure S5 D.

In this sense, we chose to validate the alternative promoter hypothesis by RT-PCR by selecting two transcripts (Smp_097280 and Smp_160450) presenting internal SL entry sites as case studies. For transcript Smp_097280 there are two main SL acceptor sites, one occurring in the outron region (Fig. 2A and C) and the other in the boundary of intron 3/exon 4 (Fig. 7A and C). As we used the SL as forward primer and a gene specific primer annealing to the fourth exon of this gene, we expected to visualize an amplification product of approximately 800 bp for the SL entry in the 5′ region of the transcript and a smaller product of ~250 bp for the SL alternative entry in the end of the third intron. Surprisingly, only the smaller band of 250 bp was amplified and no 800 bp product was seen, even with a strong amplification signal obtained when using a gene specific primer for the exon 1 of the gene (Fig. 2C), what confirms that the 5′ acceptor site is an important SLTS site in this transcript. This result suggests the existence of an alternative promoter in the intronic region upstream to the SL insertion in the exon 4. In the other case study, the transcript Smp_160450 was amplified using the SL forward primer and a reverse primer annealing to exon 17. The amplification products of ~600 bp and ~150 bp were seen in the gel, confirming that the main sites at the boundary of intron 15/exon 16 and intron 16/exon 17 were used as alternative SL entry sites and, probably, no internal promoter exists in this region, refuting the hypothesis that SLTS depends on the absence of an upstream splice donor site, in this case a cis-splicing donor site, to occur.

**Figure 8:**
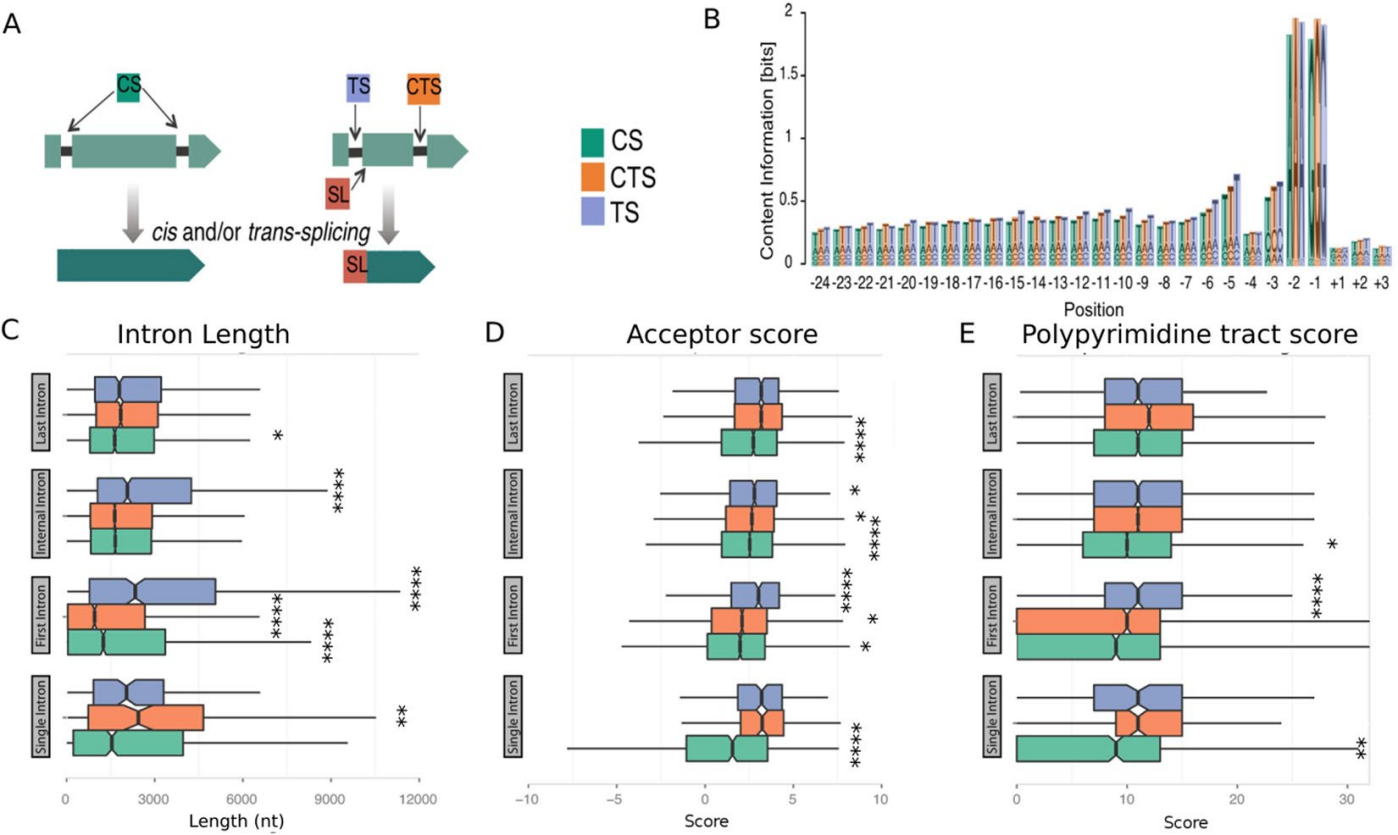
Cis and trans-splicing features based on the intron positions. (**A**) Introns were subdivided in three distinct groups for the following analysis: CS - cis-spliced introns (green boxes), CTS - cis-spliced introns in trans-spliced transcripts where the intron is not the primary SLTS target (orange boxes), and TS trans-spliced introns (purple boxes). (**B**) Intron/exons boundaries represented by the region from −24 to + 3 nucleotides. Acceptor splice sites in SL-containing genes were analysed based on their sequence composition. (**C**) Intron lengths are plotted based on their position in genes (single, first, internal, and last introns). (**D**) The strengths of acceptor splice sites (log-odds scores) are plotted according to the intron position in genes of the same three distinct groups. (**E**) Length of the polypyrimidine tract in nt relative to the acceptor sites are plotted according to intron position in genes of the same three distinct groups. Significantly differences are pointed (Kolmogorov-Smirnov test, *p < = 0.05, **p < = 0.01,***p < = 0.001, ****p < = 0.0001).

To investigate the properties that discriminate trans-spliced from non-trans-spliced introns, we employed the classification system generated by Davis and colleagues^7^ for cis-spliced introns (CS), cis-spliced introns in trans-spliced transcripts (CTS) and trans-spliced introns (TS) (Fig. 8A). We observed uridines at a higher frequency in intronic regions, proximal to the trans-splice acceptor sites, compared with cis-splice acceptor sites in trans-spliced transcripts or cis-splice acceptor sites in cis-spliced transcripts. The uridine content is even greater near to the AG splice site (Fig. 8B). By further distinguishing introns based on their annotated transcript structure into single, first, intermediate, and last introns, we observed that TS introns are typically longer (Fig. 8C), exhibit increased acceptor splice site scores (Fig. 8D) and contain longer polypyrimidine tracts (Fig. 8E). Taken together, our results show that SLTS events at intron acceptor sites are not a random observation, but exhibit particular characteristics with implications for the determination of specific splicing mechanisms.

## Discussion

We present the first in depth investigation of SLTS in *S. mansoni* based on transcriptome-wide sequencing of trans-splicing enriched genomic regions. Our analyses demonstrate that: (1) the SL Trapping is a powerful and reproducible technique that confirms earlier observations based on regular RNA-Seq data and provides an incomparably higher resolution for SLTS studies; (2) a substantially greater subset of transcripts undergo SLTS than suggested by previous studies; (3) transposons and retrotransposons might bear the SL; (4) the parasite carries twice as many polycistrons, than has been reported before; (5) it does not seem to exist a direct correlation between SLTS frequency and gene expression; (6) weaker acceptor sites for SLTS may occur upstream or downstream the main entry site, especially in the outron region; (7) there is a tendency to occur SLTS events in the first exon, however, a large amount of SLTS events take place in different introns of a same transcript, characterizing an ATS; (8) longer introns may harbor internal alternative promoters producing shorter transcripts lacking the upstream donor site, and intensify the probability of ATS; (9) in the absence of internal promoters, long introns may act as outrons due to the distance between acceptor and donor cis-splicing sites; (10) trans-splicing intron acceptor sites share particular characteristics that distinguish them from non-trans-spliced introns.

This unprecedented depth of interrogation allowed the assessment of rare SLTS events and novel trans-spliced genes that were previously below the detection threshold of standard RNA-Seq experiments, and highlight the relevance of this approach as a complementary method for enhancing genome annotations. Here, we show that at least 46% of cercariae *S. mansoni* transcripts are processed by trans-splicing. This is substantially higher than the 10% reported on previous studies focused on a small subset of genes or based on standard RNA-Seq datasets^7,20^. Our observations are consistent with similar works using focused strategies to study SLTS in the nematode *C. elegans* and in the urochordate *C. intestinalis*, in which 70%^17^ and 58%^19^ of all transcripts are reported to undergo SLTS, respectively. However, our repository of *S. mansoni* SLTS gene transcripts may be incomplete since our analyses were predominantly performed on data derived from the cercariae and expression pattern of (trans-spliced) genes are known to vary throughout the parasite life-cycle^5,26^.

We further identified 32 genes containing the SL as an internal sequence of a gene body, six of them corresponding to transposable or retrotransposable elements. These SL-containing retrotransposons might correspond to trans-spliced retro-transcribed elements, since such retrotransposons are particularly active in cercariae and schistosomula stages^27^ and therefore were observed to be expressed in our dataset. However, these findings could also reflect a chimeric assembly of the parasite reference genome, because 40% of the *S. mansoni* genome is composed of repetitive regions^28^.

Although we were able to increase the number of identified polycistrons in *S. mansoni*, these elements still constitute a minority of trans-spliced mRNAs, reinforcing that SLTS might exert another role beyond the polycistron resolution in the parasite. We also observed that most of the polycistronic units, despite being controlled by the same promoter, have different expression levels. This could be probably explained by the presence of functional sequence elements in the RNA sequence controlling RNA metabolism^29^.

Overall, our results identify SLTS as an ubiquitous RNA processing mechanism in *S. mansoni* that plays a key role in the regulation of a wide range of genes as any obvious enrichment for a gene functional category or for a chromosomal origin among the trans-spliced genes was observed, corroborating previous reports demonstrating that SLTS in *S. mansoni* are not associated with a particular tissue, developmental phase, gender, or with specific genes or gene families^5,7^. These findings are consistent with a comprehensive transcriptomic analysis in *Eutreptiella* sp. which concluded that trans-spliced transcripts are functionally diverse^30^ and supported the role of SLTS as a pervasive, ubiquitous mechanism in the parasite.

Albeit the SLTS detection could be facilitated in transcripts derived from highly expressed genes, we also observed trans-spliced transcripts derived from low expressed genes and few trans-splicing events in highly expressed genes. In *C. elegans*, a direct correlation between SLTS frequency and gene expression was demonstrated using a whole transcriptomic approach^17^, in which rare events are disregarded. By using a focused strategy, we show here that the frequency of SLTS is not directly linked to the level of gene transcription, but markedly affected by structural features in the gene body that can facilitate or hinder trans-splicing, as showed by *in vitro* studies^23,31^.

One remarkable feature revealed by our study is the location of the acceptor site. As expected, we observed a preference for SLTS events occurring in the first exon, rendering to SLTS mechanism to be more restrained in cis acceptor sites, and more permissive at acceptor sites around the 5´ end, the outron, most likely due to the absence of competition with splice donor substrates in cis. However, although the most frequent SL insertions were observed in the first exon, they represent a small portion of all SLTS events in *S. mansoni*. Moreover, our analysis revealed a substantial proportion of SLTS events occurring in different introns of a same transcript, characterized as ATS. In those cases, the trans acceptor sites coincided mostly with the annotated cis acceptor sites and, therefore, reflected the canonical splice site signals.

By investigating the presence of alternative promoters located upstream to the cis-acceptor sites used alternatively for SLTS, we identified introns that are very likely to contain independent promoters to produce alternative transcript isoforms, probably representing a not yet characterized mechanism in *S. mansoni* for producing protein diversity. Future experiments for validation of internal promoters and transcription start sites in the class of genes is crucial for understanding how their expression is regulated. Although our *in silico* analysis was very conservative and may be underestimating the actual number of alternative promoters located in introns, the majority of trans-splice sites in cis acceptor sites seems to occur in the presence of a 5′ donor site. Our suggestion that long introns may enhance the predisposition for trans-splicing is supported by other organisms findings demonstrating that internal promoters generate alternative transcripts lacking the 5′ donor site for splicing in cis^18,25^ and also that long introns can be interpreted as outrons by the splicing machinery, mainly when there is a large distance between the 5′ and 3′ splice sites^17^. The differential insertion of the SL sequence in ATS acceptor sites along the transcript body highlights possible additional roles for ATS in the parasite, i.e., protein inactivation due to the removal of the start codon or alteration of the transcript open reading frame, alteration of protein subcellular localization due to elimination of signal peptides or generation of isoforms partially or fully lacking the related domains, but these points require further investigation.

Our analysis also demonstrates that genes exhibiting comparatively higher levels of ACS isoforms display reduced trans-splicing frequency, suggesting a general competition between splicing within the same molecule and splicing between different molecules. Furthermore, genes with more exons and comparatively low ACS frequency are more subjected to SLTS, suggesting a higher efficiency of the trans-splicing, in comparison to cis-splicing, in transcripts with weak ACS sites. These observations are in agreement with other findings showing that the trans-splicing efficiency can be markedly affected by downstream elements that promote exon definition and recognition of splice-acceptor sites^23,31–33^ as well as longer and richer polypyrimidine tracts, confirming similar studies in *T. brucei*, which report that polypyrimidine tracts content can directly influence trans-splicing efficiency^34^.

Finally, we show here that several splicing features play a key role in committing an intron to cis- or trans-splicing. Thus, the spliceosome in organisms such as *S. mansoni*, where cis- and trans-splicing co-exist could be seen as a unique machinery carrying both U1 and SL snRNP, and their affinities for those features being the major determinant for selecting cis or trans-splicing events.

To the best of our knowledge, our experiments yielded the most representative and comprehensive data on SLTS in *S. mansoni* which was extensively analyzed to explore the role of SLTS in this organism. Such observations may be further extended to organisms from other taxa for which the SLTS mechanism has been characterized and may contribute to build a more thorough understanding of SLTS evolution and function in eukaryotes.

## Methods

### Dataset compilation of RNA-Seq reads from SRA

Publicly available RNA-Seq datasets from different stages of the *S. mansoni* life cycle were included in this study. The dataset characteristics are listed in Supplemental Table S4. Twelve raw files that were generated using Illumina platform were downloaded from the SRA repository^35^ and converted into the fastq format. The trans-spliced sequences were filtered according to whether the 36 nt sequence of the *S. mansoni* spliced leader was present using the program Illumina reads adapter screening utilities/sortPairedReads (with -Q 33 -f sl_smansoni.fasta -s 19 parameters). Next, the SL sequence was trimmed from the reads using the program Illumina reads adapter screening utilities/trimReads (with -f sl_smansoni.fasta -s 19 -m 0 -q 0 -Q 33 parameters); these reads were compiled in a file referred to as the RNA-Seq Filtered dataset.

### RNA isolation

The *S. mansoni* (LE strain) life cycle was maintained at the Centro de Pesquisas René Rachou (CPqRR), Fundação Oswaldo Cruz, Brazil. The total RNA from *S. mansoni* (cercariae stage harvested from the intermediate host *Biomphalaria glabrata* snails*-* Barreiro de Cima strain) was isolated using the TRIzol® Reagent (Invitrogen) and RNeasy Kit (Qiagen) and then treated with Ambion® RNase-free DNase I (Invitrogen). The RNA samples were quantified using the Nanodrop ND-1000 (Thermo Scientific), and all samples showed an A260/A280 ratio higher than 1.8. In addition, the RNA integrity was verified using the Agilent 2100 Bioanalyzer.

### RNA-Seq library preparation and sequencing

The libraries were constructed and sequenced by the FASTERIS facility (https://www.fasteris.com) following the protocol described in^15^ with the following modifications. The primer specific to the *S. mansoni* SL sequence ([BIOT]5′-AATGATACGGCGACCACCGAGATCTACACTCTTGTGATTTGTTGCATG-3′) was used to produce the second strand cDNA, and sequencing using the Illumina HiSeq2000 platform was performed with a specific sequencing primer (5′-GAGATCTACACTCTTGTGATTTGTTGCATG-3′). Two libraries from independent biological replicates were sequenced using a 1 × 100 bp run and are referred to as the SL Trapping datasets.

### Data processing and mapping of the reads to the *S. mansoni* genome

The statistics and quality analyses of the reads were generated using the FastQC software version 0.10.1^36^. To identify the SLTS targets, RNA-Seq Filtered reads trimmed by the SL sequence and SL Trapping reads were aligned to the genomic sequence of *S. mansoni* (5^th^ version), which was downloaded from the GeneDB^37^, using the TopHat2 mapper^38^ version 2.0.9 (with -i 10 -I 30000 -p 8 -library-type fr-unstranded parameters). Subsequently, all SRA RNA-Seq reads from the cercariae stage were aligned to the genome using the same approach before to estimate the expression levels of the cercariae genes based on the GeneDB transcriptome annotation (5^th^ version). The mapped reads were filtered by mapping quality using the threshold of score 20 and unique mapping using Samtools version 0.1.19^39^ (with -bhq 20 -F 0 × 100 parameters).

### Gene expression and SLTS counts normalization

The raw counts for each gene in the *S. mansoni* GeneDB gene and exon annotation (5th version) were obtained for all datasets using the HTSeq Python package version 0.5.3p3^40^ using the “–mode = intersection-strict” option for exon counting. As a cutoff, only genes/exons with ≥10 reads count and represented in both Trapping datasets were considered for further analyses. Normalization of the gene/exon counts, and their expression values was performed using the DESeq2 R package version 1.2.5^41^, including a matrix with the gene lengths for normalization.

### Functional annotation

As most of the trans-spliced genes were annotated as “hypothetical proteins”, we re-annotated the predicted mRNA sequences of *S. mansoni* gene models by employing an established pipeline described in^42^. In brief, we performed blastx, blastn, or rpsblast^43^ searches for the coding DNA sequences against several databases, including Swissprot^44^, Gene Ontology^45^, KOG^46^, Pfam^47^, and SMART^48^, and a subset of the non-redundant protein database from NCBI that contained vertebrate proteins. Further manual annotation was performed as required. The results were used to assign a functional classification to the protein sequences through the Classifier program (developed by Dr. José Marcos Chaves Ribeiro, NIAID/NIH), which is based on a vocabulary of nearly 250 keywords found in matches to all of the databases used, as well as their e-values, to produce nearly 30 functional categories. We performed a Chi-squared test (p-value < 0.05) to identify significant enrichment of a given transcript class that undergoes trans-splicing compared with the cercariae stage transcriptome. The UniProtKB/TrEMBL identifiers of *S. mansoni* protein gene models were associated with their respective counts in the datasets. The program iPath^49^ was used to generate the *S. mansoni* metabolic pathways using the KEGG database^22^, and the pathways containing trans-spliced genes were represented by red lines, with thickness reflecting the number of normalized read counts per gene.

### Identification of polycistronic units

Gene groups located in the same chromosome and DNA strand with intergenic distances of up to 200 bp (i.e., the distance from the 3′ end of the upstream to the 5′ end of the next downstream annotated gene) were detected in the *S. mansoni* gene model annotation using our customized script. We suggest the existence of *bona fide* polycistronic units through assessing the occurrence of SLTS in their downstream genes. Alleged polycistronic clusters were required to exhibit SLTS in at least one gene downstream of the first gene in the polycistronic unit. Certain predictions were visually verified using IGV 2.1 (Broad Institute)^50^.

### Identification of gene *loci* containing the SL sequence

We performed a BLAT^50^ search (with -t = dna -q = dna -minIdentity = 90 -out = blast8 –maxGap = 1 -fine parameters) on the *S. mansoni* genome for the 36 nt SL sequence. SL genes encoding the SL RNA were identified and removed from the results. After removing the SL genes, the genome was intersected with the gene model annotation to identify genes containing embedded SL sequences.

### Identification of SLTS signals

The reference gtf was used to obtain the exonic and intronic regions of the different transcripts of *S. mansoni* and used to map the acceptor sites present in both libraries on the transcript and feature (intron or exon). Sequences surrounding the splice sites where the SL sequence was inserted were retrieved from genes using BedTools version 2.17.0^52^ and visualized as sequence logos using the WebLogo 3.0 software^53^. To investigate feature changes between the cis- and trans-spliced sites, we used a conservative approach that focused on a set of trans-spliced genes with at least one trans-spliced exon at >10 counts to an internal exon and positive values in both SL Trapping libraries. In these genes, the intron with the highest count at its 3′-end was identified as the main target for trans-splicing; all other introns for the same gene were considered cis-spliced introns in the trans-spliced transcripts. Acceptor sites located in outron regions were not included in this analysis. A balanced set of genes not subject to trans-splicing but with similar expression levels yielded a comparable number of introns spliced in cis. Using these intron sets, we then employed the GeneID^54^ splice site models to score potential donor and acceptor sites, which represent the first order Markov chains trained on annotated splice sites. To assess the branch point features, we applied different models integrated into the Support Vector Machine SVM-BPfinder^55^ to the annotated intronic sequences.

### Promoter prediction

In order to evaluate the enrichment of SLTS events on transcripts produced from alternative promoters located inside the preceding intron used as SLTS target, we made use of an *in silico* approach to predict canonical promoters. The promoter prediction softwares Easy Promoter prediction Program (EP3)^56^ and Promoter 2.0^57^ were used to evaluate the presence of putative promoters genome-wide. We considered as true predictions the regions marked as ‘true’ by EP3 and also marked as ‘highly likely’ by Promoter 2.0.

### Additional *in silico* analyses

The annotation file generated was used to obtain the biotype of each gene and the corresponding gene, exon and intron lengths. All data processing and visualization was performed using R version 3.0.2^58^. Certain plots were constructed using the ggplot2 package^59^ for R, version 0.9.3.1.

### RT-PCR to validate novel trans-spliced loci and polycistrons

*S. mansoni* cercariae RNA samples were treated with RNase Free DNase Set (QIAGEN) to on-column DNA digestion followed by further treatment with RNase-free DNase I (Ambion®). RNA samples were quantified using the Qubit RNA BR Assay Kit (Thermo Scientific). cDNA was synthesized using Illustra™ Ready-to-Go™ RT-PCR Beads (GE Healthcare) and then used for PCR amplification using PCR SuperMix High Fidelity (Invitrogen). To validate novel trans-spliced loci, PCR amplifications were performed using a SL forward primer and reverse primers specific for genes with high and low counts (Supplementary Table S1). As a positive control, the gene already described as trans-spliced^60^, Smp_024110 (enolase), was used. A non-trans-spliced gene, Smp_045200 (tegument-allergen-like protein), was used as a negative control. Another subset of genes was used to validate the occurrence of trans-splicing in internal exons. PCR amplifications were performed using a forward SL primer and an exon-specific reverse primer (Supplementary Table S1). For polycistron validation, two PCR reactions were carried out as described by^20^ for each polycistron; the first PCR used gene specific primers from upstream and downstream genes to demonstrate the presence of the primary polycistronic transcript. The second PCR used the SL forward primer and a gene specific reverse primer to demonstrate the occurrence of trans-splicing (Supplementary Table S1). Controls without reverse transcriptase were used to show the absence of gDNA contamination. All PCR products were analyzed on 2% agarose gel stained with ethidium bromide and the bands were visualized under UV light.

### Availability of data

The RNA-Seq data for this study were obtained from the SRA archive; the accession numbers are described in Supplemental Table S4. The SLTS trapping experiments are available in the SRA archive, accession numbers (SRA:SRR1134198 and SRA:SRR1134204). SLTS counts for either SL Trapping and RNA-Seq Filtered can be found on Supplementary Table S5.

## Electronic supplementary material

Dataset 1

Dataset 2

Dataset 3

Dataset 4

Dataset 5

Dataset 6
