# Supplementary material for: Landscape of the spliced leader trans-splicing mechanism in Schistosoma mansoni
Source: Sci Rep. 2018 Mar 1;8:3877. doi: 10.1038/s41598-018-22093-3 (PMC5832876; doi:10.1038/s41598-018-22093-3)
Supplement: Supplementary file 6 — Dataset 6 [file 41598_2018_22093_MOESM6_ESM.doc]

## **Supplementary Figures**

**Landscape of the spliced leader trans-splicing mechanism in *Schistosoma mansoni***

Mariana Boroni^1,2^, Michael Sammeth^3,4^, Sandra Grossi Gava^5^, Natasha Andressa Nogueira Jorge^2^, Andréa Mara Macedo^1^, Carlos Renato Machado^1^, Marina Moraes Mourão^5*^ and Glória Regina Franco^1*^

**
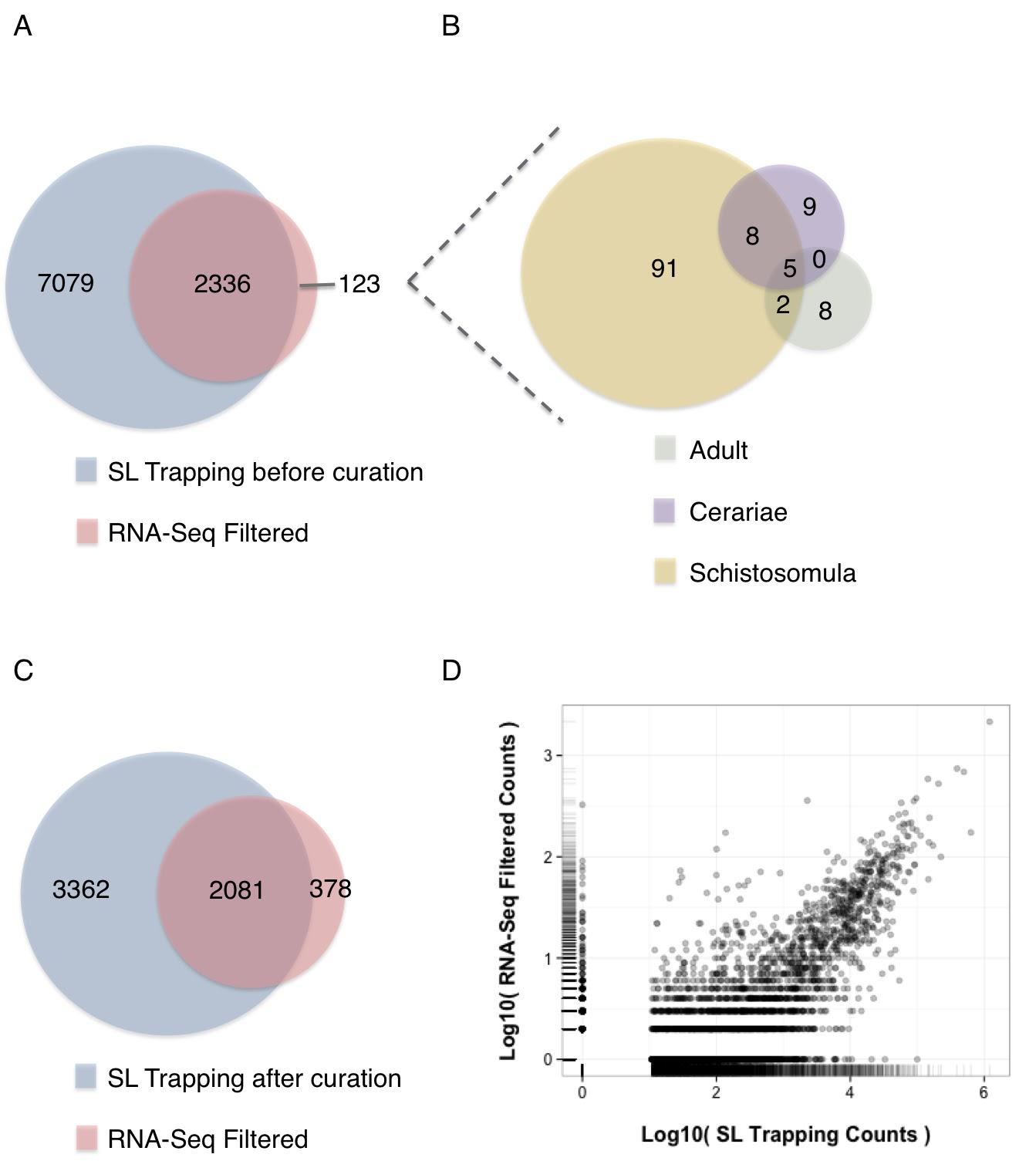
**

**Figure S1 - Comparison between the SL Trapping and the RNA-Seq Filtered datasets.**  A – A Venn Diagram representation of the SLTS events in the Trapping (blue circle) and the RNA-Seq Filtered (pink circle) datasets before the Trapping dataset curation; B – Genes exclusively detected in the RNA-Seq Filtered dataset among three compiled stages: Adult (green), cercariae (purple), and schistosomula (yellow); C – SLTS events in the Trapping (blue circle) and the RNA-Seq Filtered (pink circle) datasets after the Trapping dataset curation, i.e., only genes identified in both Trapping replicates with at least 10 reads count were considered in the final dataset. D - Expression correlation between the trans-spliced genes in the SL Trapping and RNA-Seq Filtered datasets (Pearson correlation coefficient = 0.5).


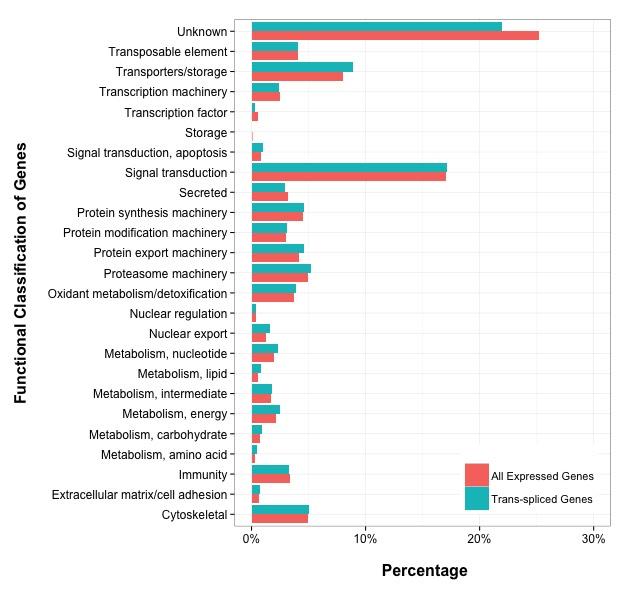


**Figure S2 – Functional classification of the trans-spliced genes.** Functional annotations for the trans-spliced genes (blue bars) are quantitatively compared with the background of all expressed genes in cercariae stage (red bars). The bars represent the percentage of transcripts classified into each functional category.


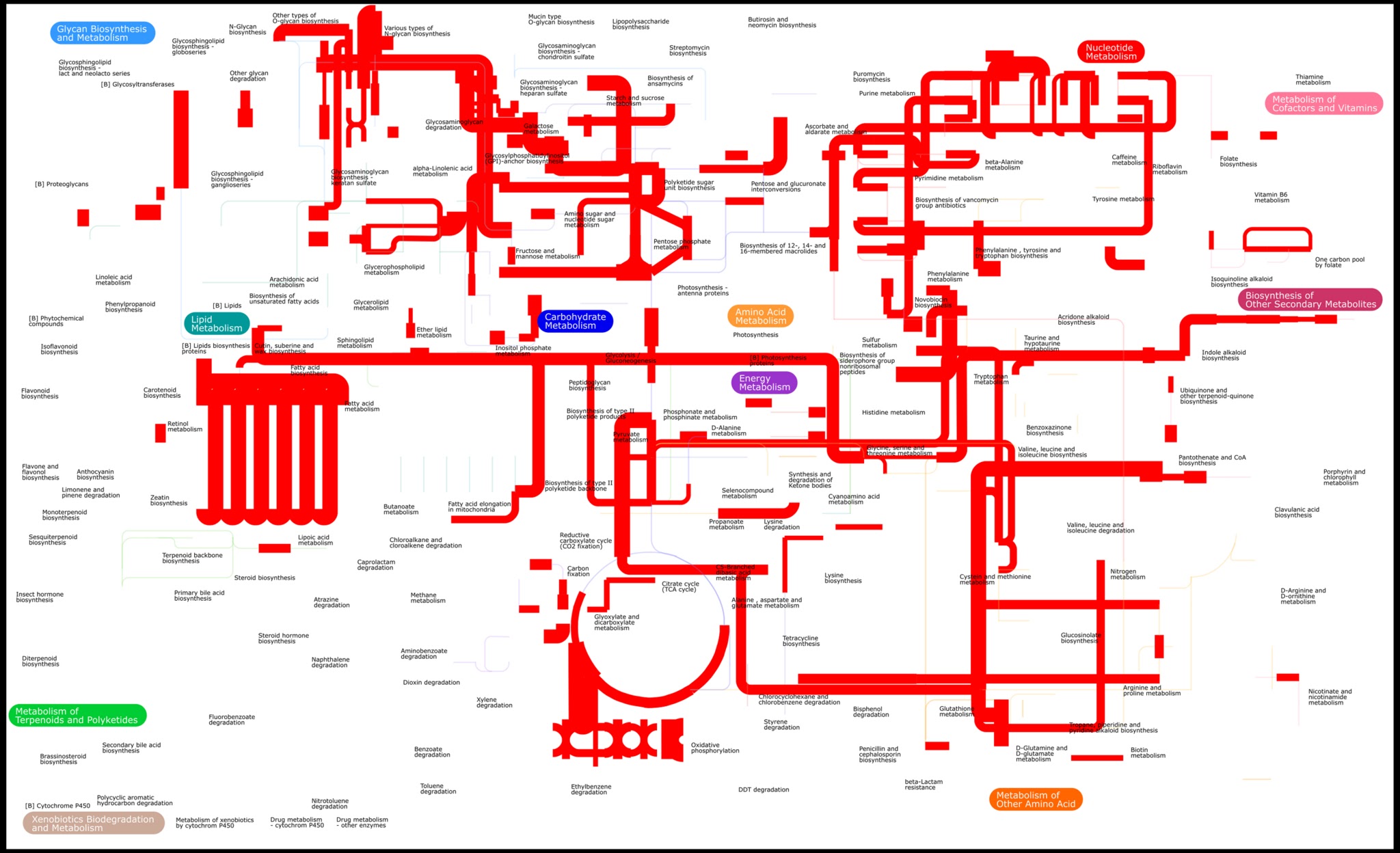


**Figure S3 – Assigned KEGG pathways ^22^ for proteins encoded by trans-spliced transcripts.** The red lines represent the metabolic pathways to which the proteins from trans-spliced transcripts are related and the line width represents the SLTS frequency.

**
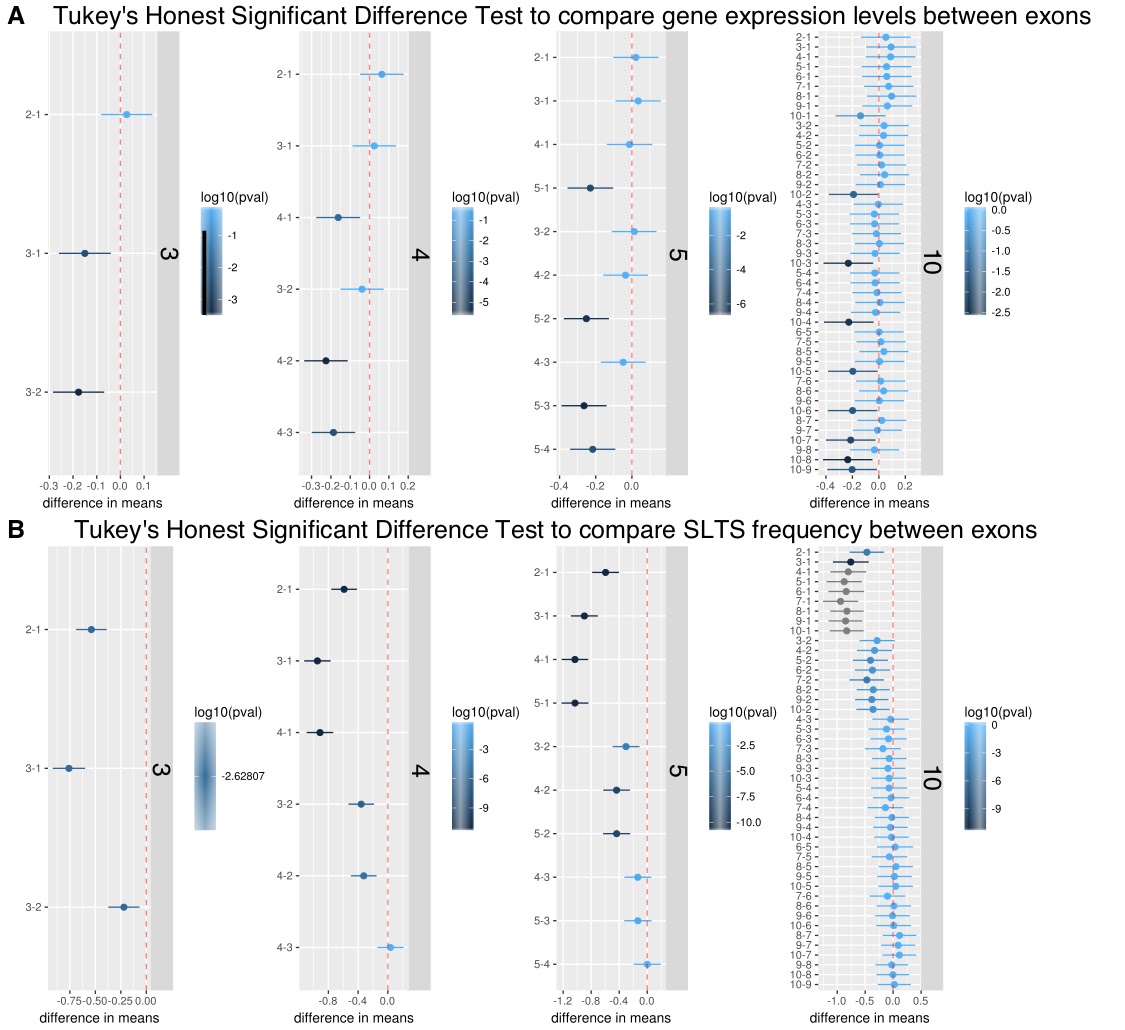
**

**Figure S4 – Tukey's Honest significant difference test.** The test was performed after ANOVA test, for the following pairwise comparisons: A - Gene expression level between exons (from Figure 5A). B- SLTS frequency between exons (from Figure 5B). Differences showing a padj less than 0.05 were considered significantly.

**
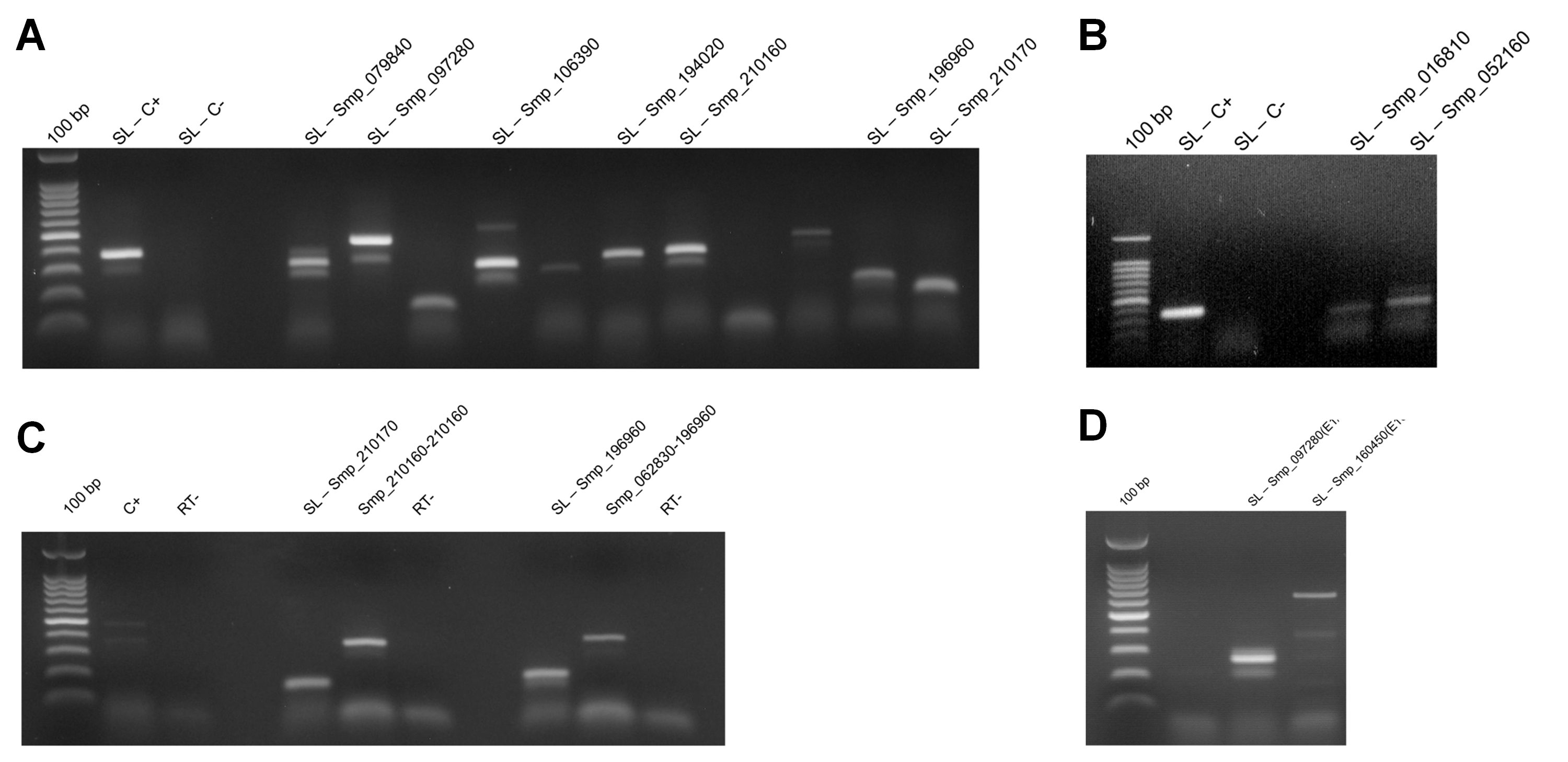
**

**Figure S5 – The full-length gels presented in Figures 2 (A, B and C) and 7 (D).**
